# Characterization and Antibiofouling Performance Investigation of Hydrophobic Silver Nanocomposite Membranes: A Comparative Study

**DOI:** 10.3390/membranes7040064

**Published:** 2017-11-12

**Authors:** Maryam Amouamouha, Gagik Badalians Gholikandi

**Affiliations:** Faculty of Civil, Water and Environmental Engineering, A.C., Shahid Beheshti University, Tehran 1658953571, Iran; m_amouha@sbu.ac.ir

**Keywords:** antibiofouling, poly(vinylidenefluoride) (PVDF), polyethersulfone (PES), silver nanoparticles, hydrophobicity

## Abstract

Biofouling is one of the drawbacks restricting the industrial applications of membranes. In this study, different thicknesses of silver nanoparticles with proper adhesion were deposited on poly(vinylidenefluoride) (PVDF) and polyethersulfone (PES) surfaces by physical vapor deposition (PVD). The crystalline and structural properties of modified and pure membranes were investigated by carrying out X-ray diffraction (XRD) and attenuated total reflectance Fourier transform infrared spectroscopy (ATR-FTIR). Scanning electron microscope (SEM) and atomic force microscopy (AFM) analyses were employed to examine the surface morphology and the bacteria anti-adhesion property of the membranes. The morphology measurements confirmed that even though after silver grafting the surface became more hydrophobic, the homogeneity increased and the flux reduction decreased after coating. Moreover a comparison between PVDF and PES revealed that CFU (colony forming units) reduced 64.5% on PVDF surface and 31.1% on PES surface after modification. In conclusion, PVD improved the performance of the membrane antibiofouling, and it is more promising to be used for PVDF rather than PES.

## 1. Introduction

Membrane separation technology has been widely used in water desalination and wastewater treatment [1,2]. Its extensive applications, however, is limited due to the occurrence of fouling [3]. Membrane fouling can be divided into organic, inorganic and biological fouling according to the foulant nature [4]. Biofouling is related to the accumulation of biomass on the membrane surface on which microorganisms mainly attach or grow [5]. Irreversible deposition and growth of microbial cells lead to biofilm formation, which will be hard to remove if enclosed in the extracellular polymeric substances (EPS) matrix [6,7]. Since biofouling results in flux reduction, and subsequently an increase in operational costs, it is essential to develop the membranes with antifouling properties [8]. In order to promote this characteristic and reduce biofouling, nanoparticles have been vastly applied, and the results have been mostly reported to be excellent [9].

For the purpose of enhancing the membrane’s biofouling resistance, two techniques are mainly employed. The first method is applying changes during the preparation process and the other method is surface modification [10]. Membrane surface modification, which is the area of interest of this study, can be realized by coating, grafting and/or deposition techniques [11]. The aim of surface modification is mainly increasing hydrophilicity, changing wettability or fabricating antimicrobial surface [12].

With the help of its antibacterial function and its minor effects on human health, the application of silver nanoparticles is a promising option to develop antifouling membranes [13,14,15,16]. Yang et al., modified an RO (reverse osmosis) membrane surface by silver nanoparticles on the membrane and the spacer. The results revealed that both coated membranes and spacer had a better antimicrobial activity in terms of more moderate flux decline [17].

Poly(vinylidene fluoride) (PVDF) has attracted attentions primarily due to its unique characteristics such as its mechanical and chemical resistant properties or thermal stability [18]. However, its application in wastewater treatment is limited by some major problems such as fouling [17]. The hydrophobicity or hydrophilicity of membranes could affect this issue [19]. It was reported by Li et al. that by increasing the nanoparticle amount, both the hydrophilicity property and antifouling function enhanced [15]. Joseph et al. concluded that the composition of nano-sized BaTiO_3_ into a PVDF matrix had better impact on shielding properties of the prepared composite rather than the micro-sized one [20]. Bio-Ag^0^, which was immobilized on PVDF, was announced to show a great virus disinfection characteristic as it decreased the virus growth by over three logs. The antiviral mechanism was reported to be Ag^+^ discharge from bio-Ag^0^. As such, the diminishing of the impact over time can limit this technology’s application [21].

An investigation of the polyethersulfone (PES) flat sheet membrane surface has been carried out by Makdissy et al. [22], in order to explore its fouling function by natural organic matter (NOM). The results revealed that the organic colloid portion was the most important factor in the flux decline. The perspective of NOM as a biopolymer mixture can contribute to an understanding of membrane fouling.

Another influential factor in the fouling issue is the surface roughness due to the impact of roughness on the surface area available to foulants. Valuable information such as quantification of fouling can be provided by the roughness measurement for the membrane surface fouling study, considering the fact that the clean membrane is often much smoother than layers of fouling particulates [23]. The study of the membrane structure impact on the fouling layer showed that this layer was further dense on the smoother membrane such as nylon [24]. 

An investigation on antibiofouling NF (nanofiltration) membranes, modified PVDF with multiwalled carbon nanotubes, expressed that the superhydrophobic membrane could reduce the influent flow and the membrane surface interaction and consequently the fouling inclination [25].

Since for both PVDF and PES the main drawback in applications is associated with the morphology and the structural properties causing fouling, this study is mainly devoted to the characterization of these factors. Although many researchers have investigated the hydrophilic modification and its effects on the membrane performance efficiency [11,15,26], less attention has been paid to the methods that create hydrophobic membranes capable of fouling reduction. In the present work, we examined the effect of hydrophobicity through physical coating on the biofouling resistance of PVDF and PES. Contact angle measurement was employed to analyze the hydrophobicity of the membrane surface. The morphology and the structure of the membranes surface have been examined via scanning electron microscope (SEM), X-ray diffraction (XRD), Fourier transform infrared (FTIR), and atomic force microscopy (AFM) analyses. The membranes antibacterial performance was investigated as well, and the results were presented in order to compare PVDF and PES.

## 2. Materials and Methods

### 2.1. Surface Modification

Silver nanoparticles at different thicknesses (i.e., 0, 15, and 30 nm) were applied to modify commercial PVDF and PES which were supplied by Millipore, Durapore^®^ (Burlington, MA, USA). The *n*Ag^0^ was coated on the membranes surface through a physical vapor deposition (PVD) method for the purpose of producing an antifouling thin film. First, the membranes were cut into 4 × 4 cm^2^, then were immersed in deionized water. Finally, the samples were put in an ultrasonic shaker for 10 min to eliminate any probable contamination. After preparation, the membrane was installed precisely in the vacuum chamber in an argon atmosphere and the pressure was reduced to 6 × 10^−5^–8 × 10^−5^ mbar with the coating rate of 0.9–1 Å/s to control the process speed. Eluding the structural destruction of the polymer, the substrate temperature must be ideally kept below ~100 °C. When the mentioned pressure was achieved, silver nanoparticles were deposited on the surface. In this process, silver is heated until it evaporates in a vacuum chamber and the metal condenses on the polymer surface. In this way, thicknesses of only a few nanometers can be realized. The thickness of the nanosilver set point was determined by SQM-160 thin film deposition monitor, Inficon (Bad Ragaz, Switzerland). The deposition time was varied in order to obtain a final thickness of 15 and 30 nm.

### 2.2. Characterization

The membrane surface hydrophilicity was observed by a contact angle meter (DSA100, Krüss, Hamburg, Germany). Distilled water was dropped onto the active layer of the surface using a microliter syringe with a flat needle, and then the contact angle was calculated. For the purpose that the experimental error was minimized, the data were collected at least three times from two different places. The mean values were reported as the contact angle. The morphology of the membranes was observed by using scanning electron microscopy (SEM) (SU-3500, HITACHI, Tokyo, Japan) combined with energy-dispersive X-ray (EDX) spectroscopy (EDS, AMTEK, Octane Prime, EDAX, Mahwah, NJ, USA). X-ray diffraction (XRD, IPDS II, STOE, Darmstade, Germany) was employed to analyze phase changes and crystalline structure of the membranes. Attenuated total reflectance Fourier transform infrared spectroscopy (ATR-FTIR) examinations were carried out by (EQINOX55, Bruker Co., Billerica, MA, USA). Prior to performing SEM/EDX and ATR-FTIR (attenuated total reflectance Fourier transform infrared spectroscopy) measurements, the membranes were dried overnight in a freeze dryer (Alpha1-2 LD Plus dryer, Martin Christ Gefriertrocknungsanlagen GmbH, Osterode am Harz, Germany). Moreover, SEM/EDX samples were coated by gold by a vacuum sputter coater (DSR1, Nanostructured Coatings Co., Tehran, Iran) before tests. The surface roughness of unmodified and modified membranes was calculated by atomic force microscopy (AFM, Model 0101/A, Ara Research Co., Tehran, Iran). Before AFM observations, the membranes were washed with ethanol/water, followed by drying at room temperature. The membrane samples were fixed on a slide glass and scanned over 5.0 μm × 5.0 μm. Obtained data was analyzed with the software of Imager 2017 (Version 1.01), provided by AFM manufacture. 

### 2.3. Antibacterial Tests

#### 2.3.1. The Colony Forming Count Methods

20 mL of Anaerobic Baffled Reactor (ABR) effluent which was diluted with peptone water was used as a mixed culture (2.3 × 10^6^ CFU/mL) for inoculation. The samples were cut into disk shape (1 cm) from both pure polymers (as controls) and their nanocomposites. First, the diluted solution was transmitted from the samples via a vacuum pump, and spread on the nutrient agar plate. Then, the samples were incubated in the plates [11,27] and finally they were put in an incubator at 37 °C for 12 h in order to count the colonies.

#### 2.3.2. Antibacterial Property

Prior to performing the tests, all nanocomposite membranes were soaked in Milli-Q water for 48 h. The anti-adhesion characteristic of the membranes were evaluated by immersion into the mixed culture effluent of ABR up to 7-day periods. The samples were then placed in a shaking incubator at stirring rate of 200 rpm and 37 °C for 1 day, 3 days, and 7 days. When the mentioned contact time spent, the membranes were directly immersed into 3% (*v*/*v*) glutaraldehyde solution for 3 h at 4 °C. The samples were post fixed in 1% osmium tetroxide for 1 h after triple rinsing with dehydrated with 30%, 50%, 70%, 90%, and 95% ethanol for 10 min in each concentration. The process was fulfilled by immersion into 100% ethanol solution twice, for 10 min [5,11]. The samples were freeze-dried overnight before performing SEM measurements.

### 2.4. Microfiltration Test

**Figure 1:**
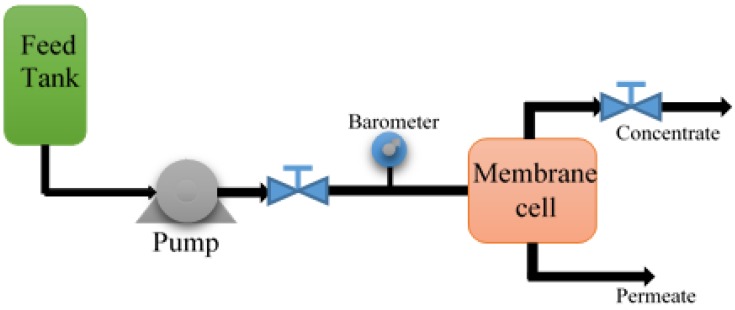
Schematic of the microfiltration setup.

A laboratory scale microfiltration unit (Figure 1) has been operated simultaneously in order to control the permeate flux reduction through modified and unmodified membranes. The membrane effective area was 16 × 10^−4^. Prior to the tests, all the membranes were pressurized with double distilled water at 0.1 MPa for 30 min. Since the effluent of ABR used as the influent of the membranes (COD: 300 mg/L, pH: 7–7.2), it was attempted to keep the operation temperature at 33–35 °C. The feed flow rate was 120 mL/min for PVDF and 700 mL/min for PES, for both the modified and the unmodified membranes.

## 3. Results and Discussion

### 3.1. Surface Hydrophilicity

The thickness of the membranes were measured using a thickness gauge at eight different places on each membrane. Then the average value and standard deviation of each one was reported in Table 1. On the other hand, as shown in Table 2, the modification via physical vapor deposition method decreased the surface hydrophilicity, which is different from the conventional plan which concentrates on development a more hydrophobic surface. The hydrophobic surface production can be related to the surface morphology and other characteristics changing through the deposition procedure. By increasing the thickness of the nanosilver deposited layer (more than 30 nm), the hydrophobicity augmented and the surface came close to superhydrophobic (i.e., contact angle is greater than 150°), which leads to the fabrication of a self-cleaned surface appearance [28]. In regard to the fouling control [10], an Ag layer thickness of 15 and 30 nm were selected to examine the efficiency of the hydrophobic nanocomposite. In this study, hereinafter, PVDF15 means PVDF membrane coated with 15 nm and PVDF30 stands for 30 nm coating on PVDF surface. This trend is applicable to modified PES membranes as well. As described in Table 2, the results showed that silver thickness is an effective parameter in the hydrophobicity. It means by coating 15 nm of silver on the polymer surface, both PVDF and PES represented the same hydrophobicity ratio. Nevertheless, deposition of 30 nm of silver caused greater hydrophobic effect on PES compared to PVDF (Table 2). This impact intensified by increasing the silver thickness. 

This finding can be justified in two different ways. First, a hydrophobic surface tends to be less bacteria captivating [29], which is applicable to both PVDF and PES. Secondly, since the hydrophobicity leads to the flux reduction, it is compatible with common sense to consider the physical coating more proper for PVDF than PES with the purpose of less flux reduction.

### 3.2. Silver Loss

To study the silver amount on the surface in different steps during antibacterial tests, EDX (energy-dispersive X-ray) measurement was employed. In this regard, images were taken from five different places on each membrane and the average was reported as the loss in Table 3. The results elucidated that in both PVDF and PES nanocomposites, increasing the silver film thickness led to a decrease in the silver release. In addition, it is obvious that the contact time had a direct impact on the silver loss. This could be explained by the point that increase in Ag content could elevate the possibility of its entrapment in the nanocomposite matrix.

Moreover, a comparison between PVDF and PES nanocomposites pointed out that the silver loss from the PES modified membrane was greater because of its higher roughness (see Table 4) and lower hydrophilicity of the surface affecting the bonding between polymer and silver. More silver loss from the PES nanocomposite membrane revealed that the silver film lifetime is less than the case with the PVDF nanocomposite. Additionally, from operational point of view, the limited silver content in permeate flow would result in restricted applications of the PES nanocomposite.

### 3.3. Membrane Surface Morphology

**Figure 2:**
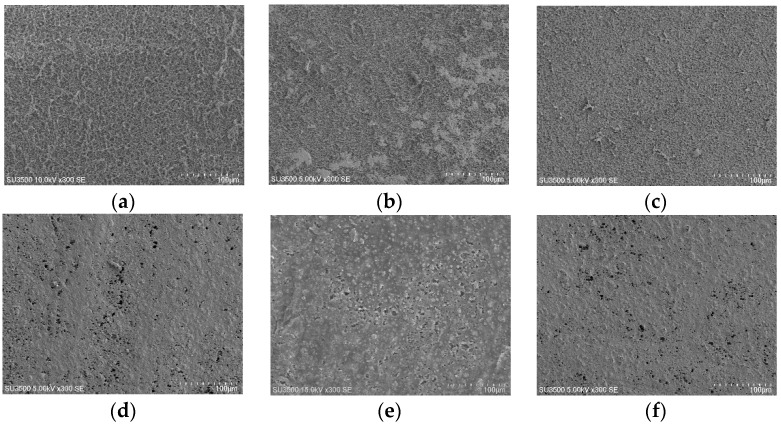
SEM images of modified and unmodified membrane surface before antibacterial test: (**a**) Pure PVDF; (**b**) PVDF15; (**c**) PVDF 30; (**d**) Pure PES; (**e**) PES15; and (**f**) PES30. Scale bar: 100 μm.

SEM was applied to investigate the modified surfaces before and after antibacterial experiments in order to evaluate the surface characteristics and the presence of Ag nanoparticles on the surface. As Figure 2 demonstrates, the nanoparticles’ distribution on the polymer matrix was approximately uniform. When comparing Figure 2b,c, it is apparent that silver was coated more monotonously in 30 nm thickness on PVDF. The same understanding could be extracted from Figure 2e,f for PES. The bigger particles’ formation could be explained as a result of heating or dissolution produced through the coating process or during the SEM measurement. In PVDF and PES nanocomposites images (Figure 2b,c,e,f, respectively), silver deposition resulted in a pore size reduction, which is in agreement with the expectation [30].

### 3.4. XRD, ATR-FTIR and AFM Results

**Figure 3:**
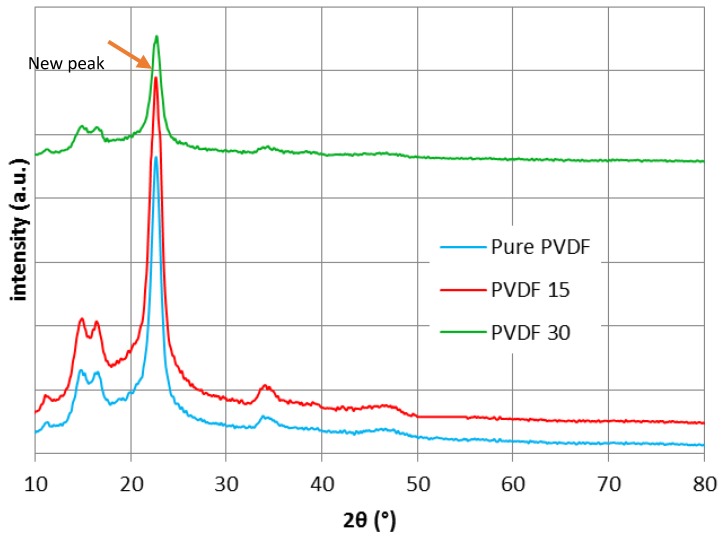
X-ray diffraction (XRD) patterns of (**a**) Pure PVDF; (**b**) PVDF15; and (**c**) PVDF30.

X-ray diffraction technique was used to appraise the structure of all pure and nanocomposite membranes during the deposition process and assess if the silver presence could make any difference in the structure or not. The diffraction angle was implied between 10° and 80° as shown in Figure 3. Generally, PVDF includes four different phases, α (form II), β (form I), γ (form III), and δ (form IV) [31]. β is responsible for the piezoelectric property, since the piezoelectricity will increase due to stronger dipole bonds during the β phase [32,33,34]. β phase development is mainly dependent on both nanoparticle size and content as well as the the interfacial bonding between the polymer and nanopaticles [35]. The XRD results verified that pure PVDF has a semicrystalline structure [36,37,38], which is a mixture of α and β fractions. The diffraction peaks are assigned to α phase emerging at 2θ = 17.8°, 18.5°, 20° and 26.8° and are allocated to *hkl* planes of (100), (020), (110) and (021) respectively [39], while the superposition of (200) and (110) characterize as β phase [34,35]. After modification, in order to develop PVDF30, the phase was transformed to β phase. This could happen due to the temperature changes during the deposition procedure, which was a reason to carry out XRD. As Figure 3 demonstrates, the appearance of a new peak next to the 22° in PVDF30 could be interpreted as the β fraction transmission. The concurrent appearance of α and β fractions caused overlapping, which could be distractive if magnification of the peaks considered as crystallinity reduction [37]. As it is obvious from Figure 3, no change emerged in the PVDF15 structure, which concords with the ATR-FTIR results. Although there is no evidence of the appearance new peaks in PVDF15, there exists a new peak, very close to the main crust, in the PVDF30 pattern. This gentle variation can be interpreted as the structural change in PVDF30. The XRD results exhibited that the silver presence eliminated some peaks from PVDF30 (around 34°) in comparison to pure PVDF. This could be justified by the silver placed among the silver chains, which increased the free volume of the polymer.

**Figure 4:**
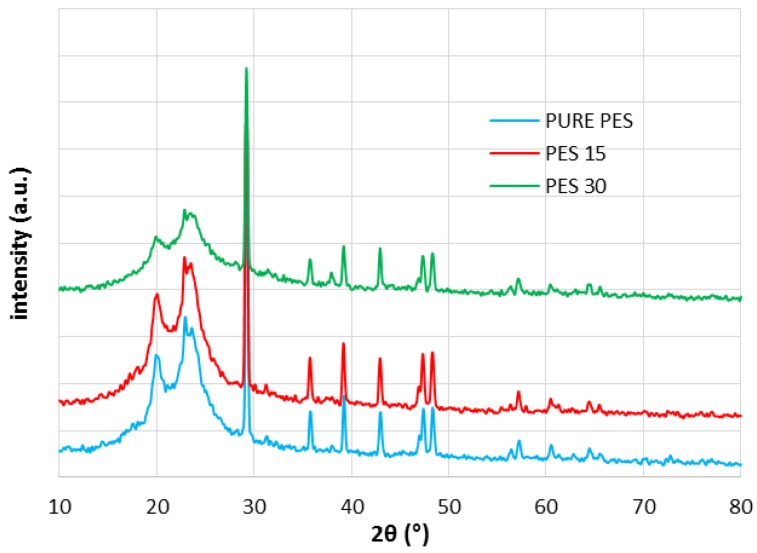
X-ray diffraction (XRD) patterns: (**a**) Pure PES; (**b**) PES15; and (**c**) PES30.

Figure 4 exhibits the XRD results for PES. As shown in Figure 4, there are four peaks for PES at 38° (111), 44.2° (200), 64.4° (220) and 77.6° (311) [6,40], which verifies the proper crystalline development of the PES nanocomposite. Like the PVDF nanocomposite, there is no evidence of β phase formation in PES15, confirming the ATR-FTIR spectra result. Altogether, the XRD results confirmed that the nanocomposite’s structure was not transformed to a more crystalline phase. 

**Figure 5:**
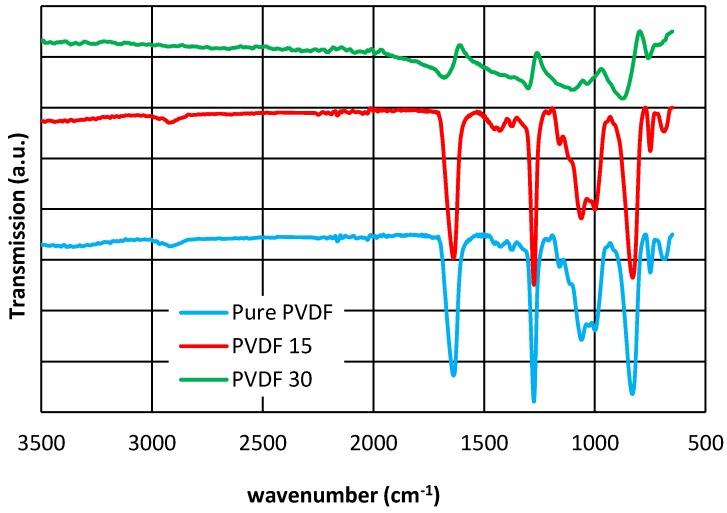
ATR-FTIR spectra of: (**a**) Pure PVDF; (**b**) PVDF15; and (**c**) PVDF30.

**Figure 6:**
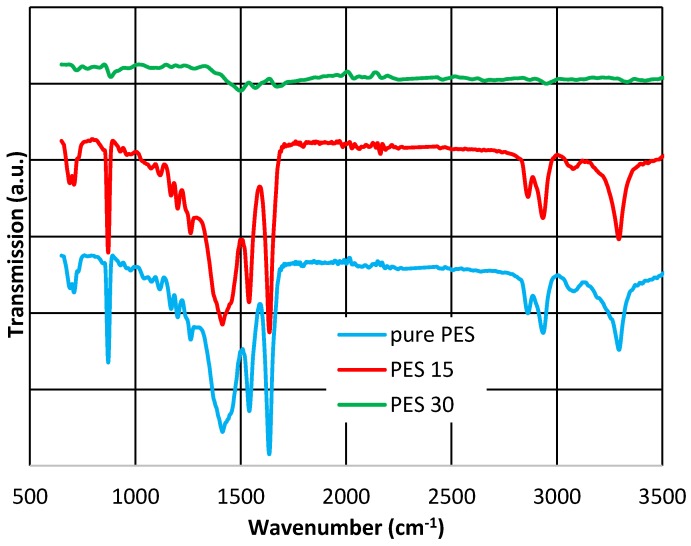
ATR-FTIR spectra: (**a**) pure PES; (**b**) PES15; and (**c**) PES30.

An ATR-FTIR spectrum was undertaken to confirm the functional group changes of PVDF and PES using infrared radiation over a range of 600 to 3500 cm^−1^ (Figure 5, Figure 6). As it has been explained in the literature, for PVDF, peaks at 840 and 1060 cm^−1^ are assigned to the CH_2_ band rocking and wagging [12,41] and belongs to the β phase. On the other hand, the band at 937 and 1275 cm^−1^, which characterizes the polymer in the α phase, are associated with the CH_2_ twisting and the CF out-of-plane deformation, respectively [35,42]. An extending band occurred at 1638 cm^−1^ due to the chemical modification happened during the silver coating, and mostly were related to the formation of the C=C double bond. This could be described as the dehydrofluorination process because of the C=O stretching vibration [43]. The decrease in intensity of the O–H band (over 2900 cm^−1^) in PVDF30 could be interpreted as a sign of hydrophilicty reduction, which also can be demonstrated by the contact angle measurement results.

The analysis of the PES membranes structure by ATR-FTIR (Figure 6) demonstrates that there is no evidence of a new functional groups formation in PES15, which is in line with the XRD results. For pure PES, the appearance of the broad band at 3294 cm^−1^ is due to the stretching vibration of hydroxyl group in PES and PES15. In the PES30 spectra, the sulfone vibration was affected by silver nanoparticles and shifted to a higher wavenumber than 1498 cm^−1^. In general, the shape of the spectrum has changed thoroughly due to the presence of silver nanoparticles.

It is also observed that as the Ag layer thickness increases, the intensity of the PES and PVDF vibrational bands increase. Apart from this, it is detected that for the both membranes as the interaction between the O–H band and Ag increases, the peak shifts to a lower wavenumber [44]. The O–H stretching vibration for pure PVDF was observed at 3382.31 cm^−1^ as Ag became attached to O–H of PVDF, the peak shifts to 3558.91 cm^−1^ for PVDF15, and to 3518.50 cm^−1^ for PVDF30. 

As shown in Figure 5 and Figure 6, it is clear that all the membranes contain similar functional groups, as they all have very similar peaks in 15 nm thickness. The chemical composition was changed after the increase in silver layer thickness to 30 nm (PVDF30 and PES30).

The ATR-FTIR results indicate that silver has created more bonding in PVDF nanocomposites compared to PES, confirming the silver loss findings. On the other words, stronger bonding appeared in PVDF (e.g., CH_2_ bonding rather than O–H).

SEM, EDX, XRD and ATR-FTIR measurements were conducted to demonstrate the successful coating of silver on PVDF and PES membranes and the results presented that even though PVD did not change the crystallinity in all membranes (Figure 3, Figure 4, Figure 5 and Figure 6), the deposition was effective as it is publicized in the SEM images (Figure 2) and the EDX results (Table 3).

**Figure 7:**
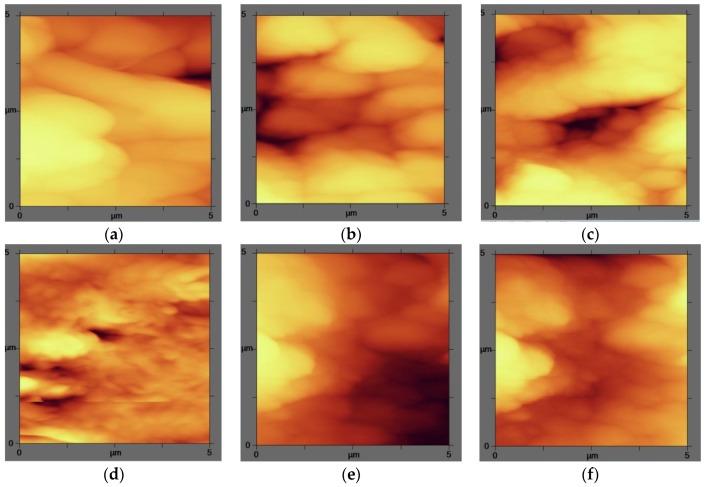
AFM plane images of the modified and unmodified membranes surfaces: (**a**) pure PVDF; (**b**) PVDF15; (**c**) PVDF 30; (**d**) Pure PES; (**e**) PES15; and (**f**) PES30 with a *Z* scale of 1 µm.

The AFM analysis was applied to determine the effect of nanoparticles on the surface roughness of the modified membranes. The images demonstrated that the nanocomposite membranes had smoother surface than the pure membranes as displayed in the plane images in Figure 7. Ag nanoparticles flattened the membrane surface; therefore the roughness decreased. Similarly, the modification made homogeneity in the nanocomposite membranes, which are less rough membranes because of the nanomaterials precipitation on the membrane surface. This is in accordance with the SEM analysis (Figure 2). As it is shown in Table 4, the effect of silver nanoparticles is greater on PVDF surfaces and the roughness has been decreased more. 

Chang et al. reported the same result after the modification of PVDF with different concentrations of PEGMA. They expressed that the RMS roughness have a dramatic decrease indicating the formation of a uniform grafted surface for the prepared membrane. However other research has revealed that the addition of the Ag nanoparticle into the polymer chain results in a growth of RMS at the first point, which is then reversed as the concentration of *n*-Ag is increased [45,46].

From all the above discussions, it can be concluded that the total surface and the structural characteristics should be argued as a set of parameters—such as the hydrophobicity and the roughness at the same time—affecting the permeation in the inner layers.

### 3.5. Antibacterial Property

#### 3.5.1. Growth Inhibition

**Figure 8:**
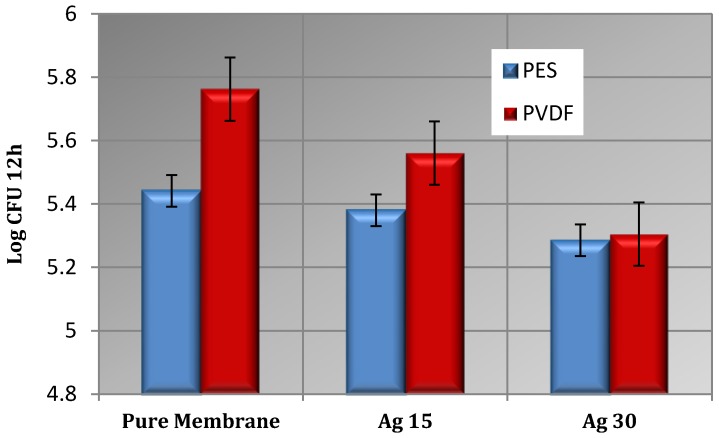
Impacts of pure and nanocomposite membranes on colony forming units counting. (Error bars were based on the standard deviations of three replicate measurements obtained from three sets of independent cultivated bacteria mixed with three independent membranes).

To investigate the efficiency of silver nanoparticles as an antibacterial agent, the numbers of colonies after 12 h have been counted and the results are shown in Figure 8. It is obvious that colonies developed more on the pure membranes surface, and the increment in the silver nanoparticles content led to a deduction in the quantity of colonies in all modified membranes, which is in agreement with the literature [38,47]. This reduction was considered as the growth inhibition effect of the silver nanoparticles amplified by a thicker silver layer. As presented in Figure 7, the antigrowth influence of the silver coating on the PVDF surface was established to be more significant than PES. In the PVDF case, log CFU (colony forming units) decreased from 5.778 (pure PVDF) to 5.322 (PVDF30) whereas in the PES case, it reduced from 5.462 to 5.301.

#### 3.5.2. Bactericidal Effect

**Figure 9:**
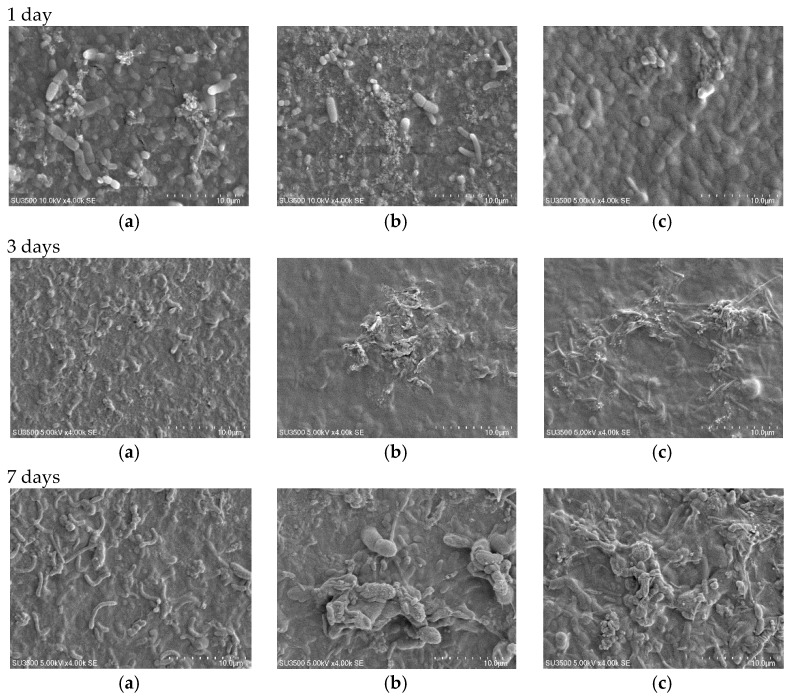
SEM images representing bacterial growth and attachment on (**a**) pure PVDF, (**b**) PVDF15, and (**c**) PVDF30 after immersion in a mixed culture from 1 day to 7 days with 4000× magnification. Scale bar: 10 μm.

**Figure 10:**
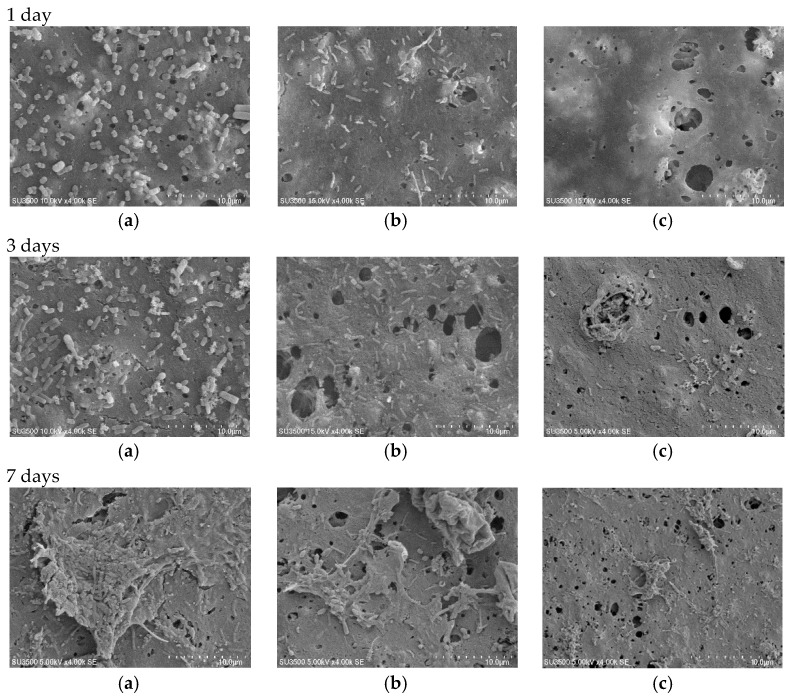
SEM images representing bacterial growth and attachment on (**a**) pure PES; (**b**) PES15; and (**c**) PES30 after immersion in a mixed culture from 1 day to 7 days with 4000× magnification. Scale bar: 10 μm.

The bacteria adhesion on the surface was detected by SEM examinations of the membranes after 1 day, 3 days, and 7 days contact with the ABR (anaerobic baffled reactor) effluent as described in Section 2.3.2. The images confirmed both the antibacterial impact and the surface modification of the nanocomposite membrane (Figure 9 and Figure 10). The images remarkably display that after one day of soaking time, the bacteria amount adhered to on pure PVDF was much greater than that of the nanocomposite membranes (PVDF15 and PVDF30). Since pure PVDF has very little bactericidal properties (Figure 9a), a larger bacteria layer developed on the surface. Comparing the modified and unmodified images in different immersion time (1 day, 3 days, and 7 days), it can be concluded that the bacteria growth on the membrane’s surface was initially high (Figure 9b,c) and it decreased due to the effect of the silver nanoparticles by rising the contact time (Figure 9c,d). After seven days, less but larger bacteria can be distinguished on pure PVDF surface, which is compatible with the literature results [5]. For PVDF15 and PVDF30, although the bacteria at the commencement of the experiment were killed by the silver nanoparticles, after 7 days of immersion, a new layer of bacteria could attach and grow on the dead bacteria corps and then a shield would be created reducing the silver nanoparticles’ antibacterial influence. Another possibility is the formation of a dead bacteria layer, which may have resulted in reduced contact between the nanosilver and the other bacteria. As it is explained earlier, the thicker nanosilver layer exhibited better antibiofouling properties, which could be justified by the smoother surface. 

Figure 10 depicts the SEM images of the modified and the unmodified PES at different contact times. Similar to the PVDF membranes, after one day of contact, the amount of the bacteria present on the pure PES surface was much more in the comparison with the PES nanocomposite (Figure 10a–c). It was even greater at three and seven days of immersion times on the pure PES surface. This could be related to the concentration of the bound EPS and lead to the more biofouling tendency. As it has been showed in Figure 10, the bacteria growth on PES15 and PES30 was less in the quantity and the membrane surface was more resistant to the biofilm formation. Even if there is a chance of a biofilm development on the surface, the modified membranes can prevent the fouling occurrence via releasing the silver ion [6,48].

According to Figure 9 and Figure 10, it could be concluded that the antibacterial effect of the modified PVDF is superior to the modified PES in terms of anti-adhesion concept. In other words, the bacteria growth rate on the modified PVDF surface (for 1 day, 3 days, and 7 days) is less than the modified PES.

### 3.6. Antibiofouling Performance

**Figure 11:**
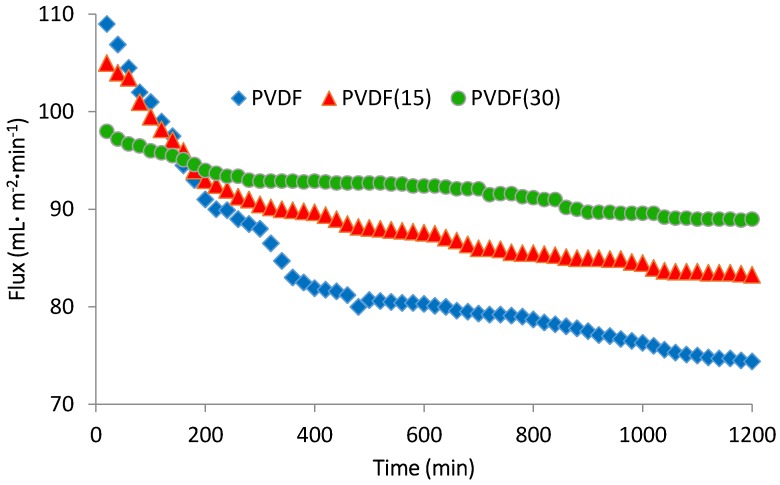
The flux variation of pure PVDF, PVDF15, and PVDF30 after 20 h filtration.

**Figure 12:**
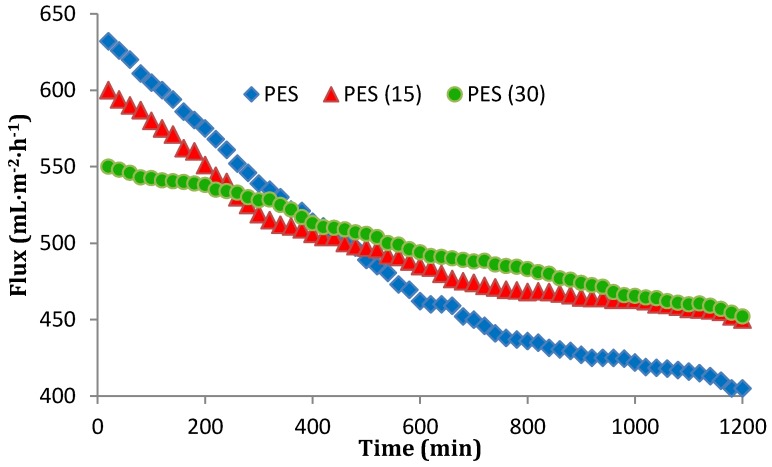
The flux variation of pure PES, PES15, and PES30 after 20 h filtration.

The flux of pure and nanocomposite membranes was shown in Figure 11 and Figure 12. The operation pressure was over the range of 0.1–0.12 MPa and the influent was provided from ABR effluent. As shown in Figure 11 and Figure 12, for both PVDF and PES unmodified membranes, the flux declined during the operating time due to the interactions between the influent and the filtration explained by the biofouling phenomenon. The modified membranes initially revealed less permeate flux (between 3.5% and 10% for PVDFs, and 5%–13% for PESs), which is acceptable owing to their hydrophobic surfaces. After an operating time of 20 h, the modified membranes flux reduction was much less than the pure membranes. In regard to this reduction, the unmodified PVDF and PES membranes experienced 31.7% and 37% flux reduction improvement, respectively. The decrease was equal to 20%, 25%, 9% and 17.8% for PVDF15, PES15, PVDF30, and PES30, respectively. These flux declines verify the conclusion that a thicker nanosilver layer leads to more antibacterial properties and therefore develops more biofouling-resistant surfaces. Another result is, although the nanocomposite membranes surface appeared to be more hydrophobic, the flux profile was acceptable and is comparable with those membranes reported to become hydrophilic during the modification [15,30]. A comparison between PVDF and PES illustrates that although PVDF is more hydrophobic (Table 2), with or without the modification, it shows a smoother flux reduction slope after 1200 min filtration time. In other words, the PVDF membranes initiated with a higher biofouling rate and a less initial flux than PES but both membranes reached a similar biofouling rate after 30 nm deposition of silver. This means the surfaces of the membranes perform the same when modified with silver. Another point which should be taken into account is the other affecting parameters on the biofouling occurrence such as the recovery rate. In this study, the recovery and other hydraulic conditions were similar for the two membranes in order to make the comparison of PES and PVDF feasible. 

## 4. Conclusions

In this work, two commercial microfiltration membranes (i.e., PVDF and PES), modified by silver nanoparticles (*n*Ag^0^) using different thicknesses of the silver layer, were studied and compared in order to have a better insight about their antibiofouling properties. Both PVDF and PES were tested under the same conditions in all the sections making comparison reasonable. Investigation of the structure of the nanocomposite membranes expressed that the permeability of the membranes is dependent not only on the contact angle, but also on the point that the silver presence could change the surface morphology. Considering these findings, the influence of the hydrophobicity on the membrane permeability should be reconsidered in order to be coupled with other factors impacting the permeability. The results of this research demonstrate that the fouling behavior can be affected by complicated factors such as surface chemical properties, pore size, hydrophilic/hydrophobic characteristic, and roughness (which could be categorized as surface morphology characteristics). These parameters should be considered together for a definite understanding of the fouling behavior in MBR processes. Another exciting result is the coating method (PVD) effect on the silver release into the effluent flow, which seems to be acceptable according to the related guidelines [49]. Lastly, the results demonstrated that the physical coating is a more proper option in terms of production of the antibiofouling specification for PVDF rather than PES. Further studies should be carried out to explore other aspects of viability of PVD as an environmentally friendly and available technique to modify membranes with the aim of wastewater reclamation.

## Figures and Tables

**Table 1:** Mean values of modified and unmodified membranes thicknesses.

Membrane Type	Thickness (µm)	Standard Deviation
Pure PVDF *	475.625	8.551
PVDF15	481.875	5.194
PVDF30	509.75	9.750
Pure PES **	64.5625	1.891
PES15	71.4625	3.062
PES30	74.3875	2.383

*: poly(vinylidenefluoride); **: polyethersulfone.

**Table 2:** Mean values of modified and unmodified membranes hydrophobicity.

Membrane Type	Contact Angle (°)
Pure PVDF	75 ± 1
PVDF15	108 ± 1
PVDF30	120 ± 1
Pure PES	61 ± 1
PES15	93 ± 1
PES30	128 ± 1

**Table 3:** Silver nanocomposite amount on modified and unmodified membranes surface measured by EDX.

Contact Time	Silver Content							
	PVDF15		PVDF30		PES15		PES30	
	Ave.	STDEV.	Ave.	STDEV.	Ave.	STDEV.	Ave.	STDEV.
Before contact	2.29	0.092	7.62	0.381	4.23	0.169	10.94	0.547
1 day	2.12	0.085	7.06	0.282	3.58	0.143	9.83	0.393
3 days	1.98	0.079	6.49	0.259	3.15	0.094	8.96	0.358
7 days	1.66	0.065	6.29	0.314	2.85	0.085	8.76	0.307

**Table 4:** Root mean square (RMS) roughness and roughness average (*R*_a_) of the modified and unmodified membranes surfaces from AFM images.

Membrane Type	RMS (nm)	*R*_a_ (nm)
Pure PVDF	228.5 ± 8	203 ± 6.3
PVDF15	196.3 ± 5.1	174 ± 4.8
PVDF30	134.9 ± 4.7	118.5 ± 4.2
Pure PES	471.1 ± 13.8	409.53 ± 11.1
PES15	358.2 ± 10.6	313.85 ± 8.7
PES30	322.81 ± 7.1	283.32 ± 4.4
